# The hidden risks of CRISPR/Cas: structural variations and genome integrity

**DOI:** 10.1038/s41467-025-62606-z

**Published:** 2025-08-05

**Authors:** Clotilde Aussel, Toni Cathomen, Carla Fuster-García

**Affiliations:** 1 Institute for Transfusion Medicine and Gene Therapy, Medical Center – University of Freiburg, Freiburg, Germany; 2 Center for Cell and Gene Therapy Freiburg (CGF), Medical Center – University of Freiburg, Freiburg, Germany; 3 German Cancer Consortium (DKTK) and German Cancer Research Center (DKFZ), Partner Site Freiburg, Freiburg, Germany; 4 Faculty of Medicine, University of Freiburg, Freiburg, Germany

**Keywords:** Genetic engineering, Genomic instability

## Abstract

CRISPR/Cas technology has revolutionized genome engineering, unlocking unprecedented therapeutic potential. However, beyond well-documented concerns of off-target (OT) mutagenesis, recent studies reveal a more pressing challenge: large structural variations (SVs), including chromosomal translocations and megabase-scale deletions, particularly in cells treated with DNA-PKcs inhibitors. These undervalued genomic alterations raise substantial safety concerns for clinical translation. As more CRISPR-based therapies progress toward the clinic, understanding and mitigating these risks is paramount. Here, we review emerging evidence on on-target aberrations and chromosomal translocations, identify key gaps in our understanding of the DNA repair pathways underlying these adverse effects, and discuss strategies to improve the safety of genome editing.

## Abstract (web summary)

CRISPR technologies hold vast potential for therapeutic gene editing, however large structural variations often arise, posing an underappreciated safety concern. Here, the authors review emerging evidence of aberrant chromosomal abnormalities and discuss strategies to improve safety outcomes.

## CRISPR editing: balancing efficiency and precision

CRISPR/Cas technology has revolutionized gene editing by enabling precise and efficient sequence-specific DNA cleavage for targeted genome modification. The system operates through a simple yet powerful mechanism: a Cas nuclease, directed by a guide RNA (gRNA), recognizes a target DNA sequence (the protospacer) via Watson-Crick base pairing, and induces a double-strand break (DSB)^1,2^. This break activates the cellular DNA damage response, leading to both intended and unintended genetic modifications. The predominant repair pathway in human cells, non-homologous end joining (NHEJ), is commonly exploited for gene knockouts, often resulting in small insertions or deletions (indels) at the cleavage site due to repeated CRISPR/Cas activity. Alternatively, co-delivery of a carefully designed DNA template can promote homology-directed repair (HDR). While HDR is less efficient than NHEJ, it enables precise sequence modifications, such as nucleotide substitutions or the insertion of large DNA fragments^3^.

Thanks to its ease of design and high efficiency, CRISPR/Cas9 has been rapidly adopted across diverse fields, from basic research to medical applications. Despite careful target site selection to minimize unintended genomic alterations, cell-based genome-wide analyses of OT activity at sites with sequence similarity to the intended target site remain crucial to evaluate the risk of genotoxic side effects. Advances in sensitive detection methods have deepened our understanding of parameters prompting OT activity, driving the engineering of Cas9 variants with enhanced target site selectivity as well as refined gRNA design^4,5^. This is particularly important given the growing clinical adoption of genome editors, exemplified by over 100 ongoing clinical trials and the recent regulatory approval of exa-cel (Casgevy®).

## Beyond indels: the complex landscape of CRISPR-induced variations

The genotoxic potential of DSBs has long been recognized, particularly in cancer biology^6^, yet early genome editing efforts largely prioritized editing efficiency over a thorough assessment of downstream genomic consequences. In recent years, however, the work of several laboratories has uncovered a more intricate picture of unintended outcomes extending beyond simple indels at OT sites. These include kilobase- to megabase-scale deletions at the on-target site^7–11^, chromosomal losses or truncations^12–17^, and chromothripsis^18^. The CRISPR/Cas system can also induce other SVs, including translocation between homologous chromosomes that results in an acentric and a dicentric chromosome^19,20^, large deletions following two cleavage events on the same chromosome^21,22^, and translocations between two different (heterologous) chromosomes, e.g. upon simultaneous cleavage of the target site and an OT site^19,23^. As any type of genomic aberration, from point mutations to large-scale chromosomal rearrangements, can ultimately lead to hazardous cellular consequences, genome-wide methods to detect such SVs have been developed. This includes CAST-Seq and LAM-HTGTS^19,23^, which were used in the pivotal studies mentioned above^7,24^. Of note, although these genomic alterations have been more extensively studied in the context of the CRISPR/Cas system, similar effects have also been observed with other DSB-inducing platforms, such as zinc-finger nucleases (ZFNs) and transcription activator-like effector nucleases (TALENs)^25,26^.

Although OT activity spans a wide range of potential consequences, it generally occurs at low frequencies and often affects functionally neutral regions or loci that reduce cell fitness, leading to their elimination by negative selection. Interpreting the biological relevance of OT edits remains difficult, but alterations in tumor suppressor genes or proto-oncogenes represent worst-case scenarios, as even rare events at these sites could drive malignant transformation. Regulatory agencies such as the European Medicines Agency (EMA) and the U.S. Food and Drug Administration (FDA) hence require a comprehensive assessment of both on-target and OT effects as well as the evaluation of structural genomic integrity to increase the safety of therapeutic gene editing applications^27^.

Despite significant advancements, the field still lacks adequate tools to assess the biological relevance of unintended edits and chromosomal aberrations. As a result, genetic evaluations rely on existing knowledge of the function of the affected gene loci. However, in cases of megabase-scale aberrations or chromosomal translocations, the impact extends beyond individual loci, affecting broader genomic regions. While OT effects have traditionally been the primary focus of safety assessments, on-target genomic aberrations deserve equal attention. The deletion of critical *cis*-regulatory elements, for instance, can have profound and unpredictable consequences. Further genotoxic consequences may result from knock-in approaches, including the unintended integration of partial or full-length DNA templates at both on-target and OT sites^28–31^. In this context, we highlight a critical concern regarding recent strategies aimed at optimizing gene editing outcomes, particularly OT mitigation approaches and HDR-enhancing methods, which may inadvertently introduce new risks.

## Balancing efficiency and risk: the pitfalls of over-tuning genome editing

The push for greater precision in genome editing has led to intense efforts to enhance HDR, the preferred pathway for precise gene modifications. Since HDR is inherently less efficient than NHEJ in human cells, researchers have explored several strategies to shift the balance toward HDR-driven repair. This includes synchronization of the cell cycle^32,33^ or small molecule drugs to inhibit key components of the NHEJ pathway like DNA-PKcs, 53BP1, or DNA ligase IV^34–39^. Fusion proteins enable a more local manipulation of DNA repair outcomes, for example by tethering NHEJ-inhibiting factors, such as dominant negative domains of RNF168 or 53BP1, to Cas9^40,41^. On the other hand, emerging studies are casting doubts on the presumed accuracy and safety of such approaches. Recent findings by Cullot et al. have unveiled that the use of the DNA-PKcs inhibitor AZD7648—a compound increasingly adopted for promoting HDR by suppressing NHEJ—can lead to exacerbated genomic aberrations^7^. The use of this compound significantly increased the frequencies of kilobase- and megabase-scale deletions as well as chromosomal arm losses across multiple human cell types and loci. Moreover, alterations were not confined to the on-target region. The OT profile was markedly aggravated, with surveys of OT-mediated chromosomal translocations revealing not only a qualitative rise in the number of translocation sites, but also an alarming thousand-fold increase in the frequency of such SVs. These results align with those of another study investigating the impact on chromosomal translocations of alternative DNA-PKcs inhibitors, further confirming that disturbing the NHEJ repair pathway alters the genomic landscape in unpredictable ways^24^.

**Fig. 1:**
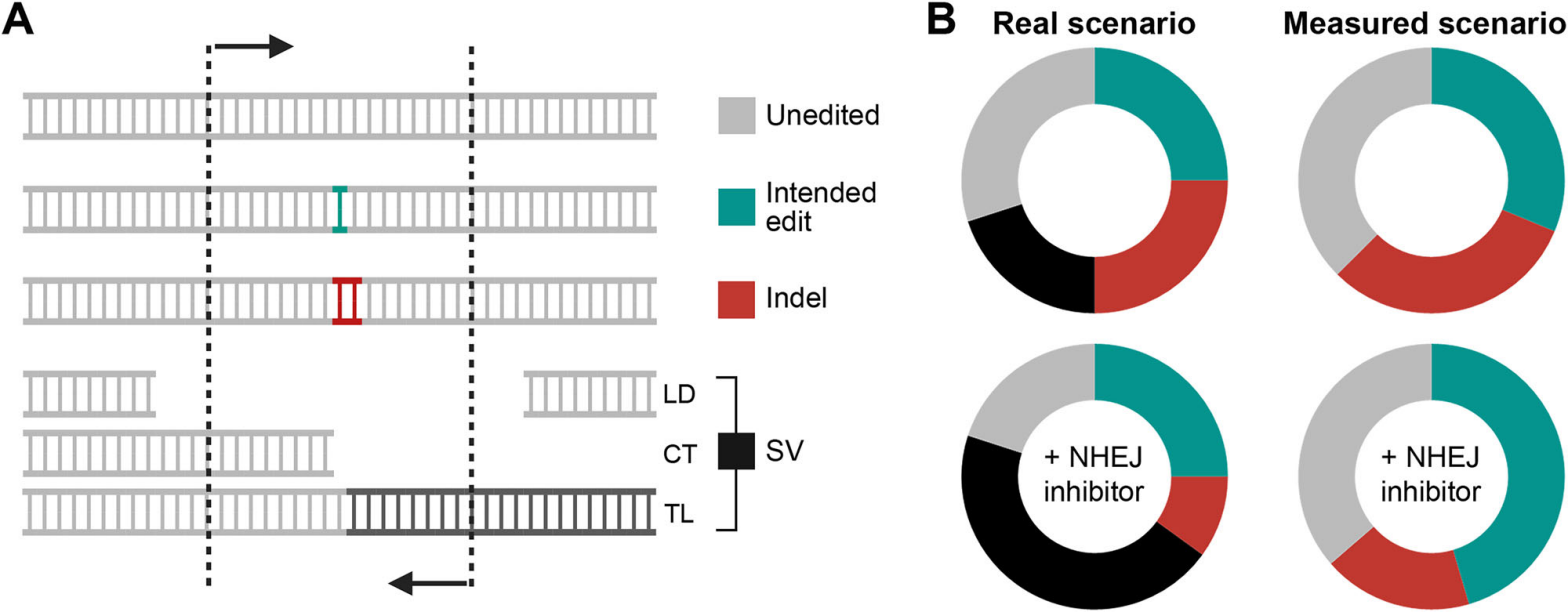
Limitations of short-read sequencing in gene editing analysis. **A** Schematic representation illustrating the limitations of traditional short-read amplicon sequencing in detecting genetic modifications beyond small indels. Different possible outcomes of an HDR-based gene editing strategy are depicted. Structural variants (SVs), such as large deletions (LD), chromosomal truncations (CT), and translocations (TL), can remove the regions where PCR primers (black arrows) bind, leading to their exclusion from sequencing analysis. Consequently, these undetected modifications introduce bias in mutation quantification. **B** Skewed quantification. Failure to account for complex genomic aberrations, e.g. by long-read sequencing, can lead to misleading conclusions. The left panel illustrates two hypothetical editing scenarios, with and without a DNA repair modulator. The right panel demonstrates how failure to detect SVs leads to an overestimation of HDR. As the fraction of undetected SVs increases, so does the miscalculation of editing outcomes, potentially compromising data interpretation in gene editing studies.

The implications of these findings extend beyond the biological risks, since they also call into question the quantitative accuracy of previously reported editing outcomes. In particular, the reported large-scale deletions would have likely misled both HDR and NHEJ quantifications. Traditional sequencing techniques based on short-read amplicon sequencing fail to detect extensive deletions or genomic rearrangements that delete the primer-binding sites, rendering them ‘invisible’ to the analysis. The consequences translate into an overestimation of HDR rates and concurrent underestimation of indels (Fig. 1). It should be noted, however, that the issues associated with DNA-PKcs inhibitors do not necessarily apply to all molecules explored for HDR enhancement. For instance, transient inhibition of 53BP1 did not affect the frequency of translocations^35^. Furthermore, co-inhibition of DNA-PKcs and DNA polymerase theta (POLQ), a key component of microhomology-mediated end-joining (MMEJ), showed a protective effect against kilobase-scale (but not megabase-scale) deletions^7,8^, although this approach has also been associated with increased loss of heterozygosity under certain conditions^17^. Of note, editing in the presence of pifithrin-α, a p53-inhibitor, was reported to reduce the frequency of large chromosomal aberrations^42^, while *TP53*-knockout increased genome instability^7^. The effect of transient p53 suppression had already been reported in earlier studies^43^, and is consistent with evidence that DSB-induced activation of the p53 pathway can trigger apoptosis, cell cycle arrest, or delayed proliferation across various cell types^43–45^. However, these same stress responses may also promote the selective expansion of p53-deficient cell clones^46,47^, raising oncogenic concerns given p53’s critical tumor suppressor role. Together, these findings underscore both the complexity of DSB repair mechanisms and the gaps in our understanding of the cellular factors that shape genome editing outcomes.

Finally, it is worth reconsidering whether increasing HDR efficiency is always necessary. In certain diseases, the corrected cells may gain a selective advantage, allowing them to expand over time^48^. In ex vivo editing contexts, post-editing selection methods can be used to enrich for successfully edited cells^49–52^. Moreover, depending on the disease, even low or moderate editing levels may be sufficient to achieve therapeutic benefit^53^.

## Off-target mitigation: when precision comes at a cost

Despite the versatility of the CRISPR/Cas system, target site constraints sometimes necessitate the use of designer nucleases with reduced specificity. To mitigate OT effects in such cases, strategies include the use of engineered Cas variants with enhanced specificity (e.g., HiFi Cas9^54^) or alternative editing approaches based on paired nicking with two Cas9 nickases (nCas9), which introduce adjacent single-strand nicks instead of a DSB through one nuclease^55^. However, while high-fidelity Cas9 variants or paired nickase strategies reduce OT activity, they still introduce substantial on-target aberrations^19,56^. Even as standalone systems—such as in base editors or prime editors—nick-based platforms may lower but do not eliminate genetic alterations^57^, including SVs^8^. These findings underscore the challenge of balancing OT suppression with genomic integrity and emphasize the need to better understand DNA damage response in CRISPR-based applications.

## CRISPR safety: clinical implications

While no medical intervention is without risks, gaps in our understanding of DNA repair mechanisms following genome editing could pose serious clinical challenges. For the first approved CRISPR therapy, exa-cel, it is well documented that targeting the GATA1 motif in intron 2 of *BCL11A* suppresses gene expression in an erythroid-specific manner, inducing fetal hemoglobin^58,59^. However, the frequent occurrence of large kilobase-scale deletions upon *BCL11A* editing in hematopoietic stem cells (HSCs) warrants closer scrutiny^9,10^. Aberrant BCL11A expression has been associated with impaired lymphoid development, reduced engraftment potential, and cellular senescence^60–62^, suggesting that cells with severely damaged chromosomes may be naturally selected against over time—potentially acting as an intrinsic safeguard. Conversely, a recent preclinical study reported impaired erythropoiesis following *BCL11A* editing in HSCs^63^, raising concerns about the long-term resilience of the graft. These findings point to possible adverse effects of *BCL11A* disruption strategies that may have eluded earlier detection or reflect limitations of current preclinical models.

Beyond these concerns, additional genetic consequences of genome editing remain poorly understood. Studies assessing OT effects, chromosomal translocations, and on-target aberrations often fail to account for critical variables such as cell type and genetic background^22^. Given that DNA repair processes vary between cell types^10^, OT analyses are best conducted in the primary cell type relevant to the intended therapy. Surrogate cell lines can be valuable—particularly when editing efficiencies are low and sensitivity is limited—and may serve a role within a classical “Nomination plus Confirmation” framework. However, it is important to recognize that such models may not fully reflect the true OT landscape, potentially introducing false positives or negatives due to epigenetic differences and genomic abnormalities such as sequence duplications, transformative mutations, or polyploidy.

Further, genetic and epigenetic differences, ethnic background, and underlying conditions—such as Fanconi anemia, which impairs DNA repair—must be considered, as they may influence both on-target and OT effects. Genomic variability presents a significant challenge, as individual polymorphisms can influence the OT profile. This is exemplified by the exa-cel case, where developers overlooked that beta-thalassemia and sickle cell disease are more prevalent in individuals of African ancestry^64^. In this population, approximately 8% carry a variant that creates a distinct OT site, which was later found to be susceptible to low-level editing^22^. Notably, the human reference genome remains the default for target design and OT evaluation, despite its limited demographic representation. Even the latest assembly does not consistently reflect the most common alleles globally, prompting calls for a transition to a more inclusive human pangenome^65^. These findings underscore the importance of using demographically representative cell sources for OT studies when feasible and support consideration of personalized assessments as part of clinical quality control strategies.

Manufacturing practices represent an additional variable that may influence genotoxic outcomes, particularly in the context of ex vivo gene-edited cells, where careful monitoring is essential. Extended culture durations and the use of proliferation-promoting reagents have been linked to higher rates of long deletions and micronuclei formation^66^, and may also elevate the risk of chromothripsis^18^.

Even clinical interventions, such as the administration of granulocyte colony-stimulating factor (G-CSF) to mitigate neutropenia during hematopoietic stem cell transplantation, have been reported to amplify the p53-mediated DNA damage response triggered by CRISPR/Cas9-induced DSBs^67^. These findings highlight the need for a more nuanced evaluation of gene-editing safety, considering both biological variability and medical protocols that may unintentionally alter genome editing outcomes.

In summary, diverse factors—including cell type, treatment modalities, and patient-specific genetic profiles—can significantly affect both the efficacy and specificity of gene editing. This underscores the importance of personalized OT assessments whenever possible, while maintaining a balanced risk-benefit perspective: even if a genetic polymorphism creates a new OT site, the therapeutic benefit may outweigh the risk. To enhance the safety and precision of therapeutic genome editing, evaluation practices should prioritize: (i) detecting complex genomic aberrations beyond point mutations, (ii) assessing on-target effects alongside OT activity, (iii) accounting for genomic complexities introduced by DNA repair modulation, and (iv) considering cell type- and patient-specific factors, including genetic background and medical interventions, that may influence editing outcomes. Ultimately, a holistic, treatment-centered evaluation of both OT and aberrant on-target effects is essential to ensure that therapeutic efficacy is not achieved at the expense of unintended consequences.
